# Senescent Human Liver Endothelial Cells Mediate CD4 ^+^ T Cell Recruitment via ICOSL

**DOI:** 10.1111/imm.70159

**Published:** 2026-06-21

**Authors:** Daniel A. Patten, Amy Lumley, Alex L. Wilkinson, Ayla O’Keeffe, Kelvin Yin, Niranjan Shirgaonkar, Ran Gao, Ramanuj DasGupta, Matthew Hoare, Shishir Shetty

**Affiliations:** ^1^ Centre for Liver and Gastrointestinal Research, School of Infection, Inflammation and Immunology, College of Medicine and Health University of Birmingham Birmingham UK; ^2^ NIHR Birmingham Biomedical Research Centre Institute of Translational Medicine Birmingham UK; ^3^ CRUK Cambridge Institute University of Cambridge Cambridge UK; ^4^ CRUK Scotland Institute Glasgow UK; ^5^ Early Cancer Institute, Hutchison Research Centre University of Cambridge Cambridge UK

**Keywords:** chronic liver disease, endothelial senescence, leukocyte recruitment, SASP

## Abstract

Senescent cells accumulate in chronically diseased liver tissues and are known to actively contribute to disease pathology. To date, these studies have predominantly focussed on senescence in epithelial cells, such as hepatocytes and biliary epithelial cells, and senescence in liver endothelial cells remains largely understudied. Here, we utilise publicly available single‐cell RNA‐sequencing data, immunohistochemical and immunofluorescent staining to detect senescent endothelial cells within chronically diseased human liver tissues. Next, we develop a novel protocol for the induction of paracrine senescence in primary human liver endothelial cells and explore their functionality. We demonstrate that senescent liver endothelial cells exhibit a reduced scavenging capacity but are still able to support lymphocyte recruitment under physiological flow conditions in vitro. Mechanistically, we determine that inducible T cell costimulator ligand (ICOSL) is an important factor in the specific recruitment of CD4^+^ T cells, but antibody blockade, genetic knockdown and genetic overexpression of ICOSL in endothelial cells has no effect on CD8^+^ T cell recruitment. Finally, we show that ICOSL gene expression is upregulated in chronically diseased tissues, present in scar‐associated endothelial cells and correlates to CD4^+^ T cell infiltration. This is the first study to demonstrate that senescent human liver endothelial cells can potentially shape the liver immune microenvironment in chronic liver disease. Targeting senescent endothelial cells could present new therapeutic opportunities to treat chronic liver diseases.

## Graphical abstract

In this study, we detect senescent endothelial cells within chronically diseased human liver tissues. Next, we develop a novel protocol for the induction of paracrine senescence in primary human liver endothelial cells and explore their functionality. We demonstrate that senescent liver endothelial cells can support lymphocyte recruitment under physiological flow conditions in vitro. Mechanistically, we determine that inducible T cell costimulator ligand (ICOSL) is an important factor in the specific recruitment of CD4^+^ T cells to the liver.

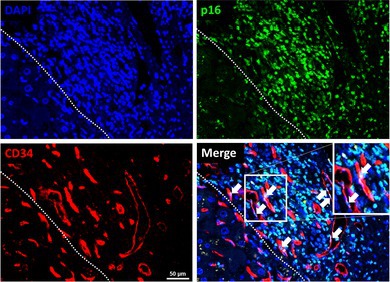

## 1 Introduction

Chronic liver diseases (CLDs) are a major cause of global mortality with the incidence continuing to rise dramatically [1]. Irrespective of aetiology, CLDs are primarily driven by persistent inflammation and, consequently, patients are at risk of serious complications, including end‐stage organ failure from cirrhosis as well as developing primary liver cancer, hepatocellular cancer (HCC) [2]. A common pathogenic pathway shared by all CLDs is the immune response to epithelial stress which leads to activation of resident hepatic immune populations and the subsequent influx of peripheral immune cells driving fibrosis and culminating in cirrhosis [3]. A key step involves the recruitment of lymphocytes across the low flow channels of the liver sinusoids, which are lined by highly specialised endothelial cells [4]. There is still a significant unmet need for effective treatments to treat liver fibrosis and HCC. Better understanding of how the liver microenvironment in CLDs shapes adaptive immune cell recruitment could have a significant impact on developing new anti‐inflammatory therapies for the treatment of cirrhosis and novel approaches to cancer prevention.

Senescence is a state of long‐term cell cycle arrest in which cells do not proliferate but remain metabolically active. There is now strong evidence that tissue senescence plays a significant role in regulating the hepatic microenvironment during CLD and the elimination of senescent cells is now being considered as a novel therapeutic approach [5]. Several key studies have implicated senescence in disease pathogenesis, with hepatocyte senescence strongly induced in both acute [6, 7] and chronic liver injury [8, 9, 10]. A hallmark of senescence is the secretion of a combination of pro‐inflammatory factors, known as the senescence‐associated secretory phenotype (SASP). The SASP is comprised of numerous cytokines, chemokines, growth factors, ECM proteins and extracellular vesicles, all of which facilitate multi‐cellular crosstalk within the senescent microenvironment [11]. Previous work has shown that SASP‐mediated activation of liver endothelial cells can regulate immune cell recruitment [12]. We have previously shown that the SASP triggers a NF‐kB‐dependent programme of lymphocyte recruitment in liver endothelial cells and found that the ICOS/ICOSL axis regulates senescent cell clearance in a murine model of oncogene‐induced senescence [13]. An additional property of the SASP is the induction of paracrine senescence in neighbouring cells [14] but, to our knowledge, there is limited understanding of how senescence induction in human liver endothelial cells impacts on their phenotype and whether they can maintain immune cell recruitment.

In this study, we aimed to evaluate how SASP‐mediated liver endothelial cell activation could be translated to human CLD. To investigate this, we initially utilised publicly available single cell RNA sequencing data to demonstrate the expression of senescence‐related genes in liver endothelial cells. Following this, we studied tissue samples of chronic human liver disease from several aetiologies and were able to detect the presence of senescence markers in scar‐associated liver endothelial cells. Next, using an in vitro approach, we demonstrated that prolonged SASP exposure can induce paracrine senescence in human primary liver endothelial cells. Additionally, these senescent endothelial cells can continue to capture and recruit lymphocytes in vitro, under conditions of physiological shear stress. Furthermore, we show that whilst CD4^+^ and CD8^+^ lymphocytes can adhere and migrate across SASP‐treated liver endothelial cells, the membrane receptor, inducible T cell costimulator ligand (ICOSL) specifically contributes to CD4^+^ T cell recruitment.

## 2 Methods

### 2.1 Human Tissue and Blood Samples

All liver tissue samples were collected from patients undergoing transplantation for chronic liver disease or rejected donor livers at the University Hospitals Birmingham (UHB) NHS Foundation Trust, with written informed consent and local ethics committee approval. Blood from patients with haemochromatosis (HFE) was collected with full consent. All experiments were performed in accordance with the regulations and guidelines sanctioned by the West Midlands—South Birmingham Research Ethics Committee, Birmingham, UK (LREC reference 06/Q2702/61 and 04/Q2708/41).

### 2.2 Publicly Available Single Cell RNA Sequencing Data

Gene expression heatmaps were generated from publicly available human liver single‐cell RNA‐sequencing data [15] via the online data browser https://shiny.igc.ed.ac.uk/Human_Liver_scRNAseq_Atlas/, accessed 02/10/2025.

### 2.3 Immunohistochemistry

Immunohistochemical staining was performed on 4 μm thick formalin‐fixed paraffin embedded tissue sections. Detailed method is presented in Supporting Information.

### 2.4 Immunofluorescence

Immunofluorescent staining was performed on 4 μm thick formalin‐fixed paraffin embedded tissue sections. Detailed method is presented in Supporting Information.

### 2.5 Liver Endothelial Cell Isolation and Culture

Liver endothelial cells were isolated from ~75 g human liver tissue as described previously [16]. Detailed method is presented in Supporting Information.

### 2.6 SASP Generation

The SASP was generated by obtaining conditioned medium (CM) from IMR90 human diploid fibroblasts undergoing oncogene‐induced senescence. This was achieved by stably transfecting IMR90 cells with a 4‐hydroxytamoxifen (4‐OHT)‐inducible form of oncogenic HRAS^G12V^ (ER:HRAS^G12V^) via the pLNCX2 ER:RAS (Addgene #67844) retroviral vector [12]. ER:HRAS^G12V^ IMR90 cells were maintained in DMEM supplemented with 10% FBS and cultured at 37°C in 5% CO_2_. Cells were then cultured in the presence or absence of 100 nM 4‐OHT (Sigma) and conditioned medium was harvested on day 6, centrifuged at 300 g for 5 min, and stored at −80°C until use. The SASP was designated ‘Ras’ and the CM from growing control cells was designated ‘Grow’.

### 2.7 SASP Treatment of Liver Endothelial Cells

Liver endothelial cells were treated with either Ras or Grow supernatants diluted in endothelial medium (1:3 ratio) for 24 h or 7 days before downstream analysis (i.e., qRT‐PCR, immunocytochemistry, flow adhesion assays). For 24 h treatment studies, cells were seeded in RTC‐coated 6‐well tissue culture plates or μ‐Slides VI 0.4 (Ibidi) and cultured overnight at 37°C in a humidified incubator with 5% CO_2_. The following day, the cells were treated with either Ras or Grow supernatants. For 7‐day treatment studies, cells were grown in RTC‐coated T75 culture flasks and treated in situ. After 6 days, cells were trypsinised and transferred to RTC‐coated six‐well tissue culture plates or μ‐Slides VI 0.4 (Ibidi).

### 2.8 Immunocytochemistry

Immunocytochemistry of liver endothelial cells was performed as described previously. Detailed method is presented in Supporting Information.

### 2.9 Quantitative Real‐Time (qRT)‐PCR

Total RNA was isolated from cell lysates using the RNeasy Micro Kit (Qiagen) in conjunction with the RNase‐Free DNase Set (Qiagen). Detailed method is presented in Supporting Information.

### 2.10 Primary Lymphocyte Isolation

Primary lymphocytes were isolated from whole blood using Lympholyte‐H (Cedarlane) and Dynabeads T Cell Kits, following the manufacturer's instructions. Detailed method is presented in Supporting Information.

### 2.11 siRNA Knockdown of ICOSL in Liver Endothelial Cells

ICOSL expression in liver endothelial cells was inhibited via small interfering (si)RNA silencing. Liver endothelial cells were transfected with 12.5 nM ICOSL Silencer Select siRNA (s225947; Thermo Fisher Scientific) or a non‐targeting siRNA control (Silencer Select Negative Control #1 siRNA; Thermo Fisher Scientific), using Lipofectamine RNAiMAX Transfection Reagent (Invitrogen). Briefly, 2.5 × 10^5^ or 7.5 × 10^4^ liver endothelial cells were seeded in rat tail collagen‐coated 6‐well cell culture plates or 0.4 Channel μ‐Slides VI (Ibidi), respectively, and cultured to confluence overnight. Subsequently, siRNA duplexes diluted in Opti‐MEM (Gibco by Life Technologies) were mixed with a final concentration of 0.3% Lipofectamine RNAiMAX and incubated for 10 min at room temperature. Cells were then washed twice with PBS and the duplex/Lipofectamine RNAiMAX mixture was added to the cells and incubated for 4 h at 37°C. The duplex/Lipofectamine RNAiMAX mixture was then removed and liver endothelial cell medium without antibiotics was added to the cells which were then maintained in standard culture conditions. After 24 h, transfected cells were stimulated with Ras or Grow supernatant and incubated for a further 24 h. Liver endothelial cells cultured in six‐well culture plates were harvested in 125 μL CelLytic MT lysis buffer with 1% Protease Inhibitor, 1% Phosphatase Inhibitor Cocktail 3 and 5 U/mL DNase‐I, and ICOSL knockdown was confirmed via Western blot analysis (see ‘Western blot’ below). Liver endothelial cells cultured in 0.4 Channel μ‐Slides VI were subsequently used in flow‐based adhesion assays (see ‘Flow‐based adhesion assays’ below).

### 2.12 Western Blot

Western blot was performed on protein lysates generated from siRNA knockdown of ICOSL in liver endothelial cells. Detailed method is presented in Supporting Information.

### 2.13 Overexpression of ICOSL in HUVEC

Human umbilical vein endothelial cells (HUVECs) were stably transfected with ICOSL using the pBABE retroviral vector [12]. The ORF of ICOSLG was obtained as pCMV3‐ICOSLG (NM_015259.4) from Sino Biological (catalogue number HG11559‐UT) and sub‐cloned into the pBabe empty vector.

### 2.14 Flow‐Based Adhesion Assays

Flow‐based adhesion assays over monolayers of primary human liver endothelial cells or HUVECs overexpressing ICOSL were used to study lymphocyte recruitment in vitro, under conditions of physiological flow, as described previously [16]. Detailed method is presented in Supporting Information.

### 2.15 Statistical Analyses

Statistical significance of all paired data was calculated via a paired *T*‐test. Statistical evaluation of data with three or more groups was performed by ANOVA with a post‐hoc Tukey's test (parametric) or Kruskall–Wallis one‐way analysis of variance with post hoc Dunn's test (non‐parametric). A *p*‐value of ≤ 0.05 was considered as statistically significant. All statistical analyses were undertaken using Prism 6 software (GraphPad Software Inc.).

## 3 Results

### 3.1 Detection of p21 and p16 Positive Scar Associated Liver Sinusoidal Endothelial Cells in Human Chronic Inflammatory Liver Disease

**FIGURE 1:**
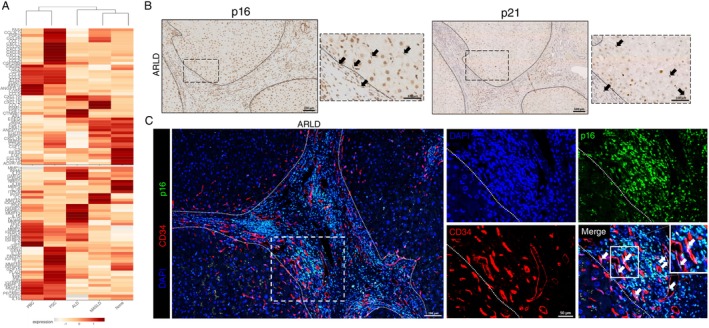
Identifying senescent endothelial cells in chronically diseased liver tissues. (A) Heatmap of the SenMayo gene signature [20] expression in endothelial cells from normal (None) and chronically diseased human liver tissues. Heatmap generated from publicly available single‐cell RNA‐sequencing data [15] via the online data browser https://shiny.igc.ed.ac.uk/Human_Liver_scRNAseq_Atlas/, accessed 02/10/2025. (B) Representative images of immunohistochemical staining (brown) for p16 (left) and p21 (right) in chronically diseased liver tissue. ARLD = alcohol‐related liver disease. Fibrotic septa are delineated with black dotted line. Black arrows indicate positively stained endothelial nuclei. (C) Representative image of multicolour immunofluorescent staining of chronically disease liver tissues. ARLD, alcohol‐related liver disease. White arrows indicate p16^+^ (green) nuclei (DAPI; blue) within CD34^+^ (red) endothelial cells.

There are several studies demonstrating the accumulation of senescent cells in human CLD; however, these have predominantly focused on epithelial changes in hepatocytes and biliary epithelial cells [8, 9, 10, 17, 18, 19]. To our knowledge, there are very few studies which characterise or explore senescence in liver endothelial cells. Using publicly available human liver single‐cell RNA‐sequencing data [15] and the SenMayo senescence gene signature [20], we demonstrate a strong senescent signature in liver endothelial cells from a range of CLD aetiologies (Figure 1A). Next, we performed immunohistochemical analysis of common senescent markers, p16 and p21, on samples of liver tissues taken from a range of CLD explants. We detected significant expression of p16^+^ and p21^+^ cells accumulating around the fibrous septa in a range of cirrhotic livers (Figure 1B, Figure S1). High magnification demonstrated that a subset of these p21^+^/p16^+^ cells had elongated nuclei which we hypothesised were endothelial nuclei (Figure 1B). To confirm that these scar‐associated senescent cells were an endothelial population, we used CD34 as a well‐recognised vascular marker which is upregulated on liver endothelial cells in chronic inflammation [21]. Dual colour immunofluorescence identified p16^+^CD34^+^ vascular cells in and around the fibrous septum and in close association with large numbers of other p16^+^ cells (Figure 1C).

### 3.2 An In Vitro Model of Prolonged SASP Exposure of Endothelial Cells Confirms Induction of Paracrine Senescence in Primary Human LSEC and Impact on Scavenging Efficiency

Given the spatial proximity of p16^+^ endothelial cells to other potentially senescent (p16^+^) cells (Figure 1B), we hypothesised that the endothelial cells may be undergoing paracrine senescence, a process in which senescence is transmitted to neighbouring cells via the senescence‐associated secretory phenotype (SASP) [14]. To explore the impact of SASP exposure on liver endothelial cell phenotype and functionality, we used the well‐established model of oncogene‐induced senescence (OIS) in IMR90 cells [12, 22] and collected the conditioned media (CM) from these cells to use as a model of the SASP. We have previously shown that 24‐h stimulation by OIS‐CM can lead to human liver endothelial activation via the NF‐kB pathway [13]. Building on this, we undertook further studies to study the impact of varying durations of OIS‐CM exposure on liver endothelial phenotype and structure. We found that acute (< 24 h) OIS‐CM exposure leads to time‐dependent morphological and cytoskeletal changes (Figure S2) and an increase in scavenging activity (Figure S3A). To mimic the chronic disease setting and explore whether a more prolonged exposure to OIS‐CM would have distinct effects on liver endothelial cells compared to acute stimulation, we undertook a model of prolonged SASP exposure over 7 days. In contrast to the 24‐h stimulation, we observed a decrease in scavenging function in 7‐day SASP treated cells (Figure S3B), which was also associated with a reduction in gene expression of several key scavenger receptors (Figure S3C).

**FIGURE 2:**
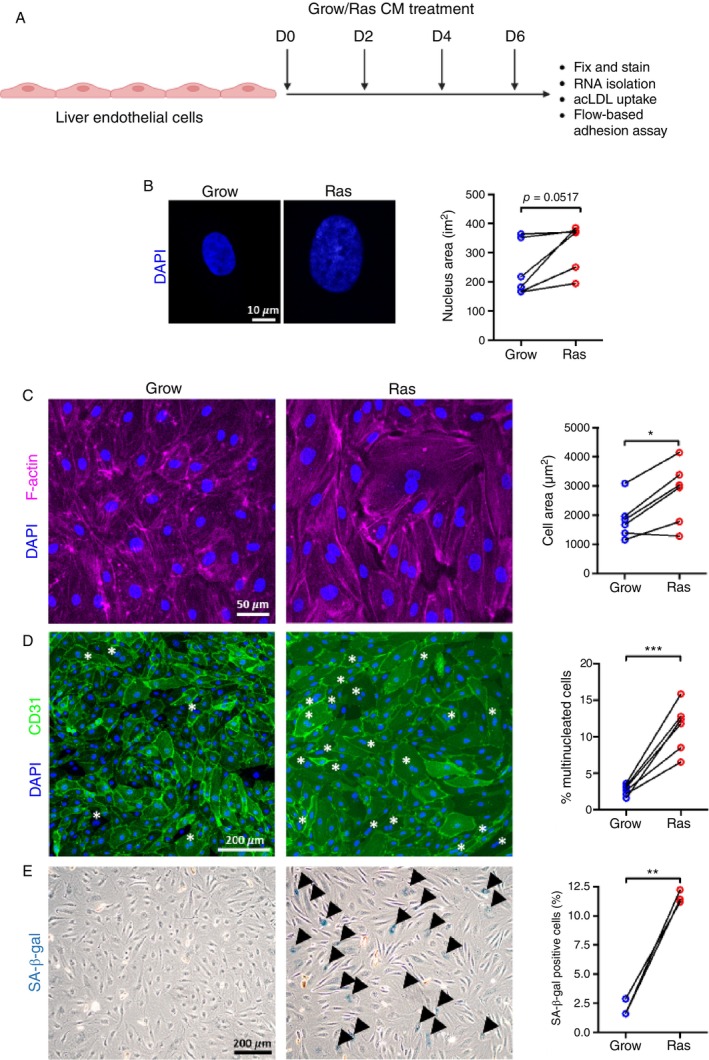
Inducing paracrine senescence in primary human liver endothelial cells. (A) Schematic of experiment design. (B) Nuclear area, (C) Cell area and (D) Multinucleation in liver endothelial cells treated with SASP (Ras) or growing cell control supernatant (Grow) for 7 days. (B) Nuclei labelled with DAPI (blue). (C) Filamentous Actin labelled with phalloidin AF633 (magenta). * indicates statistical significance, where *p* ≤ 0.05 (D) Cell area delineated by CD31 staining (green). Multinucleated cells are indicated by white asterisks. *n* = 6 independent donors with five fields of view analysed for each. *** indicates statistical significance, where *p* ≤ 0.005. (E) Senescence‐associated β‐galactosidase (SA‐β‐gal) (blue) accumulation in liver endothelial cells with SASP (Ras) or growing cell control supernatant (Grow) for 7 days. Positive cells are indicated by black arrowheads. *n* = 3 independent donors with five fields of view analysed for each. ** indicates statistical significance, where *p* ≤ 0.01.

Importantly, we also found evidence that prolonged SASP exposure can induce paracrine senescence in cultured human liver endothelial cells (Figure 2). Liver endothelial cells treated with 7 days of SASP demonstrated several features that characterise senescent cells, including a trend towards larger nuclei (Figure 2A), increased cell area (Figure 2B), increased incidence of multinucleation (Figure 2C) and increased senescence‐associated beta‐galactosidase (SA‐β‐gal) positivity (Figure 2D).

### 3.3 Senescent Liver Endothelial Cells Can Support Leukocyte Capture and Recruitment Under Conditions of Flow

**FIGURE 3:**
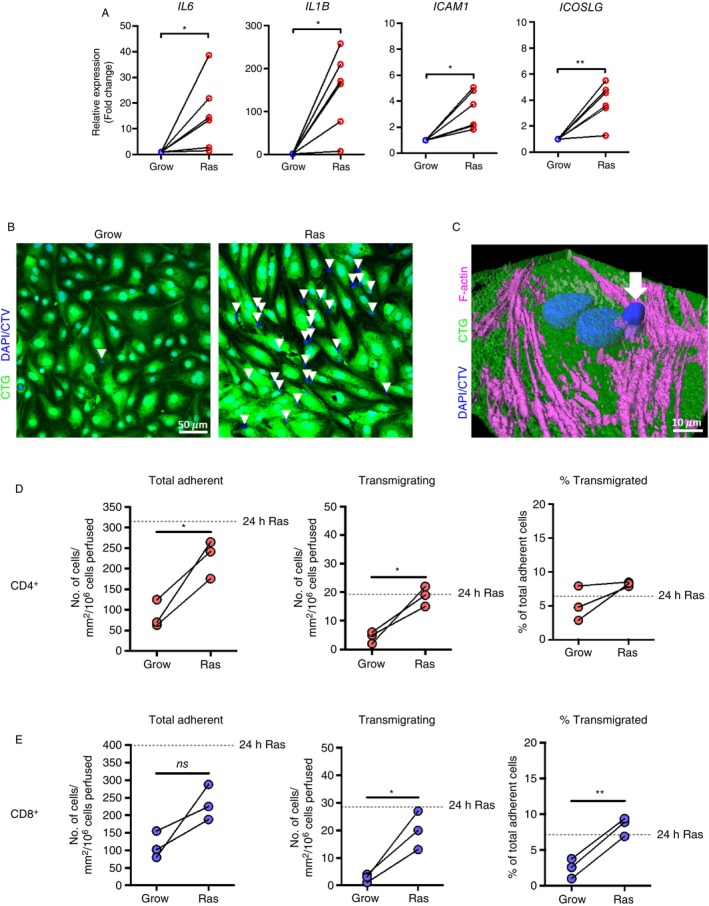
Liver endothelial cells undergoing paracrine senescence exhibit an inflammatory phenotype and support recruitment of lymphocytes under physiological flow in vitro. (A) qPCR of endothelial activation genes in liver endothelial cells with SASP (Ras) or growing cell control supernatant (Grow) for 7 days. *n* = 6 independent donors. * and ** indicate statistical significance, where *p* ≤ 0.05 and *p* ≤ 0.01, respectively. (B) Representative images of peripheral blood lymphocytes (PBLs; blue; Cell Trace Violet (CTV)‐labelled and DAPI stained) adhered to 7‐day SASP‐stimulated (Ras) or growing cell control (Grow)‐treated liver endothelial cells (green; Cell Tracker Green (CTG)‐labelled). White arrow heads indicate an adhered PBL. (C) 3D rendered *z*‐stack of an F‐Actin‐enriched adhesive cup (magenta; phalloidin AF633‐labelled) mediating the transcellular transmigration of a peripheral blood lymphocyte (blue; Cell Trace Violet (CTV)‐labelled and DAPI stained) through an enlarged and binucleated, 7‐day SASP‐treated liver endothelial cell (green; Cell Tracker Green (CTG)‐labelled). White arrow indicates transmigrating PBL. Recruitment of isolated (D) CD4+ and (E) CD8+ T cells to 7‐day SASP‐stimulated (Ras) or growing cell control (Grow)‐treated liver endothelial cells. *n* = 3 independent donors with 12 fields of view analysed for each. Dotted line indicates level of recruitment to 24 h Ras‐treated liver endothelial cells. * and ** indicate statistical significance, where *p* ≤ 0.05 and *p* ≤ 0.01, respectively.

Despite their loss of scavenging function (Figure S3B), 7‐day SASP treated liver endothelial cells maintained an upregulation of several inflammatory genes, such as *IL6*, *IL1B*, *ICAM1* and *ICOSLG* (Figure 3A). Building on our findings of senescent liver endothelial cells in chronic inflammatory liver disease (Figure 1), we aimed to explore whether these cells could contribute to immune cell recruitment to the liver. We initially repeated our model of prolonged SASP exposure to induce senescence in liver endothelial cells and then undertook flow‐based adhesion assays [16] with peripheral blood lymphocytes (PBLs; Figure 3B,C). Consistent with our previous findings that acute (24 h) SASP treated liver endothelial cells can support lymphocyte recruitment [12, 13, 23], we confirm that 7‐day SASP treatment also induced PBL recruitment when compared to control supernatant treated cells (Figure 3B). Furthermore, confocal microscopy highlighted the transcellular transmigration of PBLs through multinucleated senescent liver endothelial cells (Figure 3C). This model permits the perfusion of immune cells across endothelial monolayers at a shear stress that reflects the liver sinusoids (0.05 Pa); using phase contrast and confocal microscopy, each step of the adhesion cascade can be visualised [16]. Using these assays, we confirmed the capture, firm adhesion and transmigration of isolated CD4^+^ and CD8^+^ lymphocyte subsets across our senescent human liver endothelial cells (Figure 3D,E). The level of CD4^+^ and CD8^+^ lymphocyte recruitment to 7‐day SASP treated liver endothelial cells was comparable to that observed with acute (24 h) SASP treated cells (Figure 3D,E). To our knowledge, this is the first demonstration that senescent human liver endothelial cells can mediate immune cell recruitment and potentially regulate the immune microenvironment in liver tissue.

### 3.4 ICOSL Mediates the Recruitment of CD4 ^+^ T Cells in CLD

**FIGURE 4:**
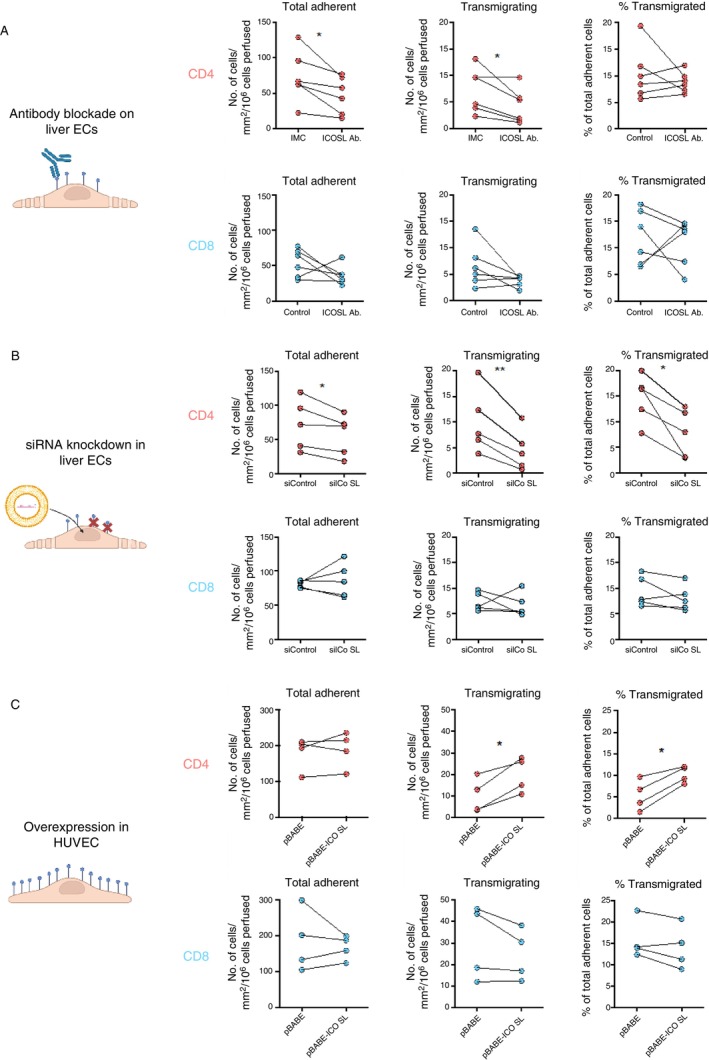
ICOSL supports the transmigration of CD4^+^, but not CD8^+^ T cells across Ras‐treated endothelial cells under physiological flow conditions in vitro. (A) Antibody blockade and (B) siRNA knockdown of ICOSL in flow‐based adhesion assays with primary CD4+ (red dots) and CD8+ T cells (blue dots), on 24 h SASP (Ras)‐treated liver endothelial cells. *n* = 5–6 independent donors with 12 fields of view analysed for each. * and ** indicate statistical significance, where *p* ≤ 0.05 and *p* ≤ 0.01, respectively. (C) HUVEC transduced to overexpress ICOSL (pBABE‐ICOSL) show increased levels of CD4^+^ T cell transmigration, when compared to vector control cells (pBABE), but no effect on the levels of CD8^+^ T cell recruitment is observed. *n* = 4 independent donors with 12 fields of view analysed for each. *indicates statistical significance, where *p* ≤ 0.05.

To provide a further mechanistic insight into how senescent human liver endothelial cells may potentially shape the immune microenvironment, we focused on the co‐stimulatory molecule inducible T‐cell co‐stimulator ligand (ICOSL). In our previous in vivo work, we have highlighted the ICOS‐ICOSL axis as an important regulator of senescence‐mediated recruitment of immune cells [13]. ICOSL is well established as a co‐stimulatory molecule; however, more recently its endothelial expression has been shown to mediate the recruitment of lymphocytes [24, 25]. We therefore assessed its contribution to immune cell recruitment in the setting of SASP‐treated liver endothelial cells (Figure 4). We used three separate methods to determine whether endothelial ICOSL contributed to lymphocyte recruitment. We utilised our flow‐based adhesion assays in the setting of ICOSL inhibition using a blocking antibody (Figure 4A) as well as genetic knockdown with siRNA (Figure 4B; Figure S4). With both these approaches we demonstrated that ICOSL inhibition specifically inhibited CD4^+^ lymphocyte recruitment in the setting of SASP stimulated liver endothelial cells but had no significant effect on CD8^+^ lymphocyte recruitment (Figure 4A,B). Additionally, we also studied the impact of gain of function of ICOSL using a HUVEC cell line overexpressing ICOSL (Figure 4C). With this approach we demonstrated that ICOSL overexpression promoted transmigration of CD4^+^ T cells in the context of SASP stimulation, but once again had no impact on CD8^+^ T cells (Figure 4C).

**FIGURE 5:**
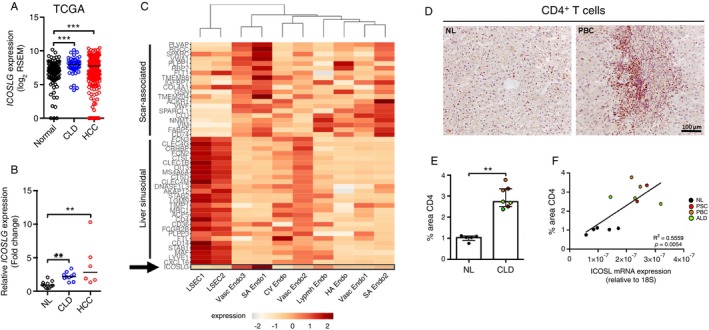
*ICOSLG* is upregulated in CLD and correlates with CD4^+^ T cell infiltration. (A) The Cancer Genome Atlas (TCGA) data showing that *ICOSLG* is upregulated in chronic liver disease (CLD) and hepatocellular carcinoma (HCC) tumour tissues, when compared to normal liver. *** indicates statistical significance, where *p* ≤ 0.005. (B) qPCR of *ICOSLG* in normal liver (NL), chronic liver disease (CLD) and hepatocellular carcinoma (HCC) tumour tissues. ** indicates statistical significance, where *p* ≤ 0.01. (C) Heatmap of scar‐associated liver endothelial cell and liver sinusoidal endothelial cell gene signature [21] expression in endothelial cell subsets from human liver tissues. Heatmap generated from publicly available single‐cell RNA‐sequencing data [15] via the online data browser https://shiny.igc.ed.ac.uk/Human_Liver_scRNAseq_Atlas/, accessed 02/10/2025. (D) Immunohistochemical staining of CD4^+^ T cells in normal liver (NL; *left*) and primary biliary cholangitis (PBC; *right*). (E) Quantification of % area positive for CD4 immunohistochemical staining shown in (D). (F) Correlation between *ICOSLG* expression and CD4^+^ T cell infiltration in normal liver (NL), primary sclerosing cholangitis (PSC), primary biliary cholangitis (PBC) and alcohol‐related liver disease (ARLD).

To further explore the pathological relevance to human disease, we accessed publicly available data sets of human chronic liver disease and hepatocellular cancer (HCC) and found that *ICOSLG* is upregulated in both diseased cohorts (Figure 5A). We validated this finding with our own cohort of samples from these three groups (Figure 5B). Next, we attempted to visualise ICOSL within CLD tissues via immunohistochemical staining; however, despite trying multiple anti‐ICOSL antibodies, we were unsuccessful (data not shown). Therefore, we once again utilised publicly available single‐cell RNA‐sequencing data [15] to confirm transcriptional expression of ICOSL in various endothelial cell subtypes (Figure 5C). This analysis demonstrated highest *ICOSLG* expression in both vascular and scar‐associated endothelial cell populations (Figure 5C). Given that our flow‐based adhesion assays had shown that ICOSL specifically mediated the recruitment of CD4^+^ T lymphocytes, we next explored CD4^+^ expression in normal liver and chronically diseased liver tissues via immunohistochemical staining. Here, we showed that CD4 expression is significantly upregulated in chronic liver disease in comparison to normal liver tissues (Figure 5D,E) and exhibits a strong positive correlation with *ICOSLG* expression (Figure 5F).

## 4 Discussion

Senescence is a cellular process that has been well established as a marker of ageing but has also been shown to play an important role at much earlier stages of life, that is, embryonic development [26]. Senescence is primarily characterised by stable growth arrest and the release of inflammatory secretome, known as the senescence‐associated secretory phenotype (SASP) [27]. In the last few years, experimental evidence has shown that the accumulation of senescent cells is also a key preservative response to cellular damage and seen in a wide range of inflammatory conditions, such as inflammatory bowel disease (IBD) [28], chronic kidney disease [29], Alzheimer's disease [30] and osteoarthritis [31]. These findings, along with in vivo functional studies, have demonstrated that cellular senescence is not simply a bystander process but actively contributes to the tissue microenvironment and can regulate inflammatory disease progression and cancer development [32]. There is therefore a significant amount of interest in the potential of targeting senescence for both therapeutic and preventative strategies.

Senescence has been implicated in both acute and chronic liver injuries, as a critical response to cell stress. How epithelial senescence impacts on cellular crosstalk is still poorly understood in the human liver. In this study, we have shown the presence of p16^+^/p21^+^ liver endothelial cells in samples of human liver disease, particularly at sites of inflammatory/fibrotic damage in CLDs. To identify endothelial cells, we used CD34, which is primarily a marker of haematopoietic progenitor cells. However, in the context of the liver, single cell studies have shown its endothelial‐specific upregulation in scar‐associated endothelium [21] and it is also utilised clinically as a tumour endothelial marker in the diagnosis of hepatocellular carcinoma [33]. Plasmalemma vesicle‐associated protein (PLVAP) and Duffy antigen receptor for chemokines (DARC/ACKR1) have also been identified by single cell studies as markers of scar‐associated endothelium in CLDs [21] and we have previously linked PLVAP endothelial expression to the level of hepatic senescence [23]. We hypothesised that senescent endothelial cells accumulated following a process of paracrine senescence by prolonged exposure to the SASP produced by surrounding senescent epithelia. We successfully recapitulated this process in vitro with primary human liver endothelial cells; interestingly, the duration of SASP exposure had a significant impact on the scavenging function of these cells. Acute exposure to SASP drives an upregulation of scavenging function and more prolonged exposure to SASP leads to paracrine senescence and a loss of scavenging efficiency. A recent murine study demonstrated the accumulation of p16^high^ senescent liver sinusoidal endothelial cells (LSECs) with age and found a similar bimodal effect on scavenger receptor expression and scavenging efficiency. In their study, Grosse et al. showed that p16^+^ LSEC taken from 12‐month‐old mice had enhanced detoxifying function which was then significantly diminished in p16^+^ LSEC taken from 24‐month‐old mice [34]. This provides new insights into how senescence impacts on liver endothelial cell function, with an acute upregulation of senescence markers being beneficial for liver endothelial cell function but persistent senescence leading to a loss of homeostatic functions. This loss of scavenging function could not only have implications for the ageing liver itself but could also have systemic consequences due to a subsequent build‐up of scavenger receptor ligands in the blood. Indeed, this phenomenon has been shown previously in a model of healthy ageing in rats, with an increase in serum malondialdehyde and bacterial lipopolysaccharide (LPS) [35].

We next studied the impact of human liver endothelial cell senescence on leukocyte recruitment. We and others have previously confirmed that the liver is a unique organ for leukocyte recruitment, with a limited role for classical selectin adhesion molecules and the contribution of atypical adhesion molecules such as vascular adhesion protein (VAP)‐1 [36, 37, 38], CD44 [39, 40] as well as scavenger receptors [41, 42, 43, 44]. We proceeded to demonstrate that senescent human liver endothelial cells can continue to recruit peripheral blood lymphocytes under conditions of physiological flow. We also show that whilst both CD4^+^ and CD8^+^ T cells can adhere and transmigrate across SASP treated liver endothelial cells, the recruitment mechanisms of these subsets are distinct. Antibody inhibition and genetic knockdown of endothelial ICOSL had a significant impact on CD4^+^ T cell recruitment, whereas CD8^+^ T cells were unaffected. Further work is required to define why ICOSL preferentially mediates the recruitment of CD4^+^ T cells in comparison to CD8^+^ T cells. It is unlikely that this is due to ICOS/ICOSL interactions as ICOS is expressed on both CD8^+^ and CD4^+^ T cells [45]. One explanation could be that endothelial ICOSL potentially contributes to the specific route of transmigration, promoting transcellular migration (i.e., through the body of the endothelial cell), as opposed to migration through the paracellular route (at endothelial junctions). Figure 3C confirms that lymphocytes can transmigrate through a senescent liver endothelial cell via the transcellular route and we, and others, have shown that CD4^+^ T cell subsets preferentially migrate via the transcellular route across endothelia [41, 44, 46] In contrast, other studies have demonstrated that CD8^+^ T cell migration across endothelia is dependent on the expression of specific junctional molecules, thus inferring a paracellular route [47, 48].

In summary, our findings have implications for lymphocyte recruitment in situations where senescent LSEC may accumulate that is, during ageing and in the context of progressive CLD. Here we provide further evidence that targeting liver endothelial senescence could significantly shape the immune microenvironment within the liver and provide new therapeutic opportunities to prevent liver fibrosis and cancer development. Specifically, our data suggest that targeting endothelial ICOSL could be a promising approach to regulate the balance of CD4^+^ T cell subsets, such as Th17 or regulatory T (T_reg_) cells, which are associated with human inflammatory liver disease, whilst maintaining CD8^+^ T cell immune surveillance against pathogens/malignant cells.

It is important to highlight some of the limitations of our study. Firstly, due to our focus on using primary human liver endothelial cells, we were dependent on access to human tissue for biological repeats rather than technical repeats, therefore impacting on experimental sizes. Larger group sizes would further support reproducibility and reduce inter‐donor variability. Nevertheless, the response of individual donor cells to SASP treatment was generally consistent, especially with regards to the significant upregulation of *ICOSL* expression. Secondly, we propose the induction of in vitro liver endothelial senescence in our model of SASP treatment, and we have attempted to use multiple markers which have previously been used to support the establishment of cellular senescence. These include cellular and nuclear area, SA‐β‐gal positivity (Figure 2), and gene signature (Figure 3A), but we acknowledge the fact that there is no single marker or factor that definitively confirms cellular senescence [49]. Additionally, many factors that are thought to promote senescence are factors which induce cellular stress, including replicative stress and oncogene‐induced stress, and previously published studies have used pro‐inflammatory cytokines or hydrogen peroxide treatment [50]. It is therefore important to differentiate between cell stress responses and cellular senescence. We were careful to ensure prolonged SASP exposure of our liver endothelial cells, as we have previously shown that 24‐h exposure of SASP clearly activates liver endothelial cells, promoting the expression of adhesion molecules and supporting leukocyte recruitment [12, 13, 23], but 24‐h exposure of SASP does not induce features of cellular senescence, such as increased cellular and nuclear area (Figure S2). We therefore confirmed clear transition to senescence only after prolonged exposure to the SASP by a combination of morphological changes and cell autonomous upregulation of factors that are contained in the SASP (IL‐1β and IL‐6), alongside the more senescent‐specific markers, SA‐β‐gal.

An additional limitation was the technical issues of detecting ICOSL protein by immunohistochemical staining in liver tissue, despite previously confirming its presence via western blotting [13]. We assume this was largely due to technical issues in unmasking appropriate epitopes in paraffin embedded tissue. Therefore, further analysis of ICOSL mRNA expression in liver endothelial cells by in situ hybridization, as well as single‐cell spatial transcriptomics to complement the single‐cell RNA‐sequencing data, may be alternative approaches for future studies.

## Author Contributions

D.A.P., M.H. and S.S. conceived the study. D.A.P., A.L., A.L.W., A.O., K.Y., N.S. and R.G. performed the investigation and formal analysis. D.A.P. and S.S. wrote the original draft of the manuscript. D.A.P., R.D., M.H. and S.S. reviewed and edited the manuscript. D.A.P., R.D., M.H. and S.S. supervised the study, were the project administrators, and acquired the funding.

## Funding

D.A.P., M.H. and S.S. are supported by a Medical Research Council project grant (MR/R010013/1). D.A.P., A.L., A.O. and S.S. are funded by a Cancer Research UK Advanced Clinician Scientist Fellowship (C53575/A29959) awarded to S.S. D.A.P. is supported by a Birmingham BRC SAFE Pre‐Application Support Fund. A.L.W. is funded by a Wellcome Trust PhD studentship in Mechanisms of Inflammatory Disease and a follow‐on fund awarded by the University of Birmingham. This research is funded by the National Institute for Health and Care Research (NIHR) Birmingham Biomedical Research Centre (BRC). The views expressed are those of the author(s) and not necessarily those of the NIHR or the Department of Health and Social Care.

## Ethics Statement

All participants provided written informed consent in accordance with the principles of the Declaration of Helsinki. All experiments were performed in accordance with the regulations and guidelines sanctioned by the West Midlands—South Birmingham Research Ethics Committee, Birmingham, UK (LREC reference 06/Q2702/61 and 04/Q2708/41).

## Conflicts of Interest

M.H. is a consultant for Quotient Therapeutics, AstraZeneca and Boston Scientific and has received unrestricted scientific grants from Pfizer. The remaining authors declare no conflicts of interest.

## Supporting information


**Figure S1:** p16 and p21 staining in chronic liver disease tissues. Representative images of immunohistochemical staining (brown) for p16 (left) and p21 (right) in chronically diseased liver tissues. MASH, metabolic‐associated steatohepatitis; PBC, primary biliary cholangitis. Fibrotic septa are delineated with black dotted line. Black arrows indicate positively stained endothelial nuclei.
**Figure S2:** Time‐dependent morphological and cytoskeletal changes in primary human liver endothelial cells in response to acute SASP treatment. Primary human liver endothelial cells were treated with Grow supernatant for 24 h or Ras supernatant for 1, 2, 4, 8 or 24 h and were fixed in 4% paraformaldehyde (PFA). Following fixation cells were imaged via phase contrast microscopy (*top*) or confocal microscopy (*bottom*). Nuclei were labelled with DAPI (blue) and filamentous actin labelled with phalloidin AF633 (magenta).
**Figure S3:** Altered scavenging capacity of SASP‐treated primary human liver endothelial cells. (A) *Left* Primary human liver endothelial cells pretreated with Ras or Grow supernatants for 24 h were treated with 10 μg/mL fluorescently labelled acetylated low‐density lipoprotein (Dil‐acLDL) for 2 h and fixed in 4% paraformaldehyde (PFA). *Right* Mean fluorescence intensity (MFI) and % area positivity were measured. ** indicates statistical significance, where *p* ≤ 0.01. (B) *Left* Primary human liver endothelial cells pretreated with Ras or Grow supernatants for 7 days were treated with 10 μg/mL fluorescently labelled acetylated low‐density lipoprotein (Dil‐acLDL) for 2 h and fixed in 4% paraformaldehyde (PFA). *Right* Mean fluorescence intensity (MFI) and % area positivity were measured. ** indicates statistical significance, where *p* ≤ 0.01. (C) qPCR of scavenger receptor genes in liver endothelial cells with SASP (Ras) or growing cell control supernatant (Grow) for 7 days. *n* = 6 independent donors. ** and **** indicate statistical significance, where *p* ≤ 0.01 and *p* ≤ 0.001, respectively.
**Figure S4:** siRNA knockdown of ICOSL in primary human liver endothelial cells. (A) Representative western blot of ICOSL and β‐actin housekeeping protein in primary liver endothelial cells treated with siRNA knockdown of ICOSL (siICSOL) or Silencer Select Negative Control (siControl). (B) Quantification of ICOSL expression, normalised to the level of β‐actin expression and expressed relative to the Silencer Select Negative Control (siControl) treated cells. *n* = 3 independent experiments with different liver endothelial cells. ** indicates statistical significance, where *p* ≤ 0.01.

**Data S1:** Supporting Information.

## Data Availability

The data that support the findings of this study are available from the co‐corresponding author (D.P. email: d.a.patten@bham.ac.uk), upon reasonable request.
