# Supplementary material for: Senescent Human Liver Endothelial Cells Mediate CD4 + T Cell Recruitment via ICOSL
Source: Immunology. 2026 Jun 21;179(1):149–60. doi: 10.1111/imm.70159 (PMC13431846; doi:10.1111/imm.70159)
Supplement: Supplementary file 1 — Figure S1: p16 and p21 staining in chronic liver disease tissues. Representative images of immunohistochemical staining (brown) for p16 (left) and p21 (right) in chronically diseased liver tissues. MASH, metabolic‐associated steatohepatitis; PBC, primary biliary cholangitis. Fibrotic septa are delineated with black dotted line. Black arrows indicate positively stained endothelial nuclei. Figure S2: Time‐dependent morphological and cytoskeletal changes in primary human liver endothelial cells in response to acute SASP treatment. Primary human liver endothelial cells were treated with Grow supernatant for 24 h or Ras supernatant for 1, 2, 4, 8 or 24 h and were fixed in 4% paraformaldehyde (PFA). Following fixation cells were imaged via phase contrast microscopy (top) or confocal microscopy (bottom). Nuclei were labelled with DAPI (blue) and filamentous actin labelled with phalloidin AF633 (magenta). Figure S3: Altered scavenging capacity of SASP‐treated primary human liver endothelial cells. (A) Left Primary human liver endothelial cells pretreated with Ras or Grow supernatants for 24 h were treated with 10 μg/mL fluorescently labelled acetylated low‐density lipoprotein (Dil‐acLDL) for 2 h and fixed in 4% paraformaldehyde (PFA). Right Mean fluorescence intensity (MFI) and % area positivity were measured. ** indicates statistical significance, where p ≤ 0.01. (B) Left Primary human liver endothelial cells pretreated with Ras or Grow supernatants for 7 days were treated with 10 μg/mL fluorescently labelled acetylated low‐density lipoprotein (Dil‐acLDL) for 2 h and fixed in 4% paraformaldehyde (PFA). Right Mean fluorescence intensity (MFI) and % area positivity were measured. ** indicates statistical significance, where p ≤ 0.01. (C) qPCR of scavenger receptor genes in liver endothelial cells with SASP (Ras) or growing cell control supernatant (Grow) for 7 days. n = 6 independent donors. ** and **** indicate statistical significance, where p ≤ 0.01 and p ≤ 0.001, respect [file IMM-179-149-s001.docx]

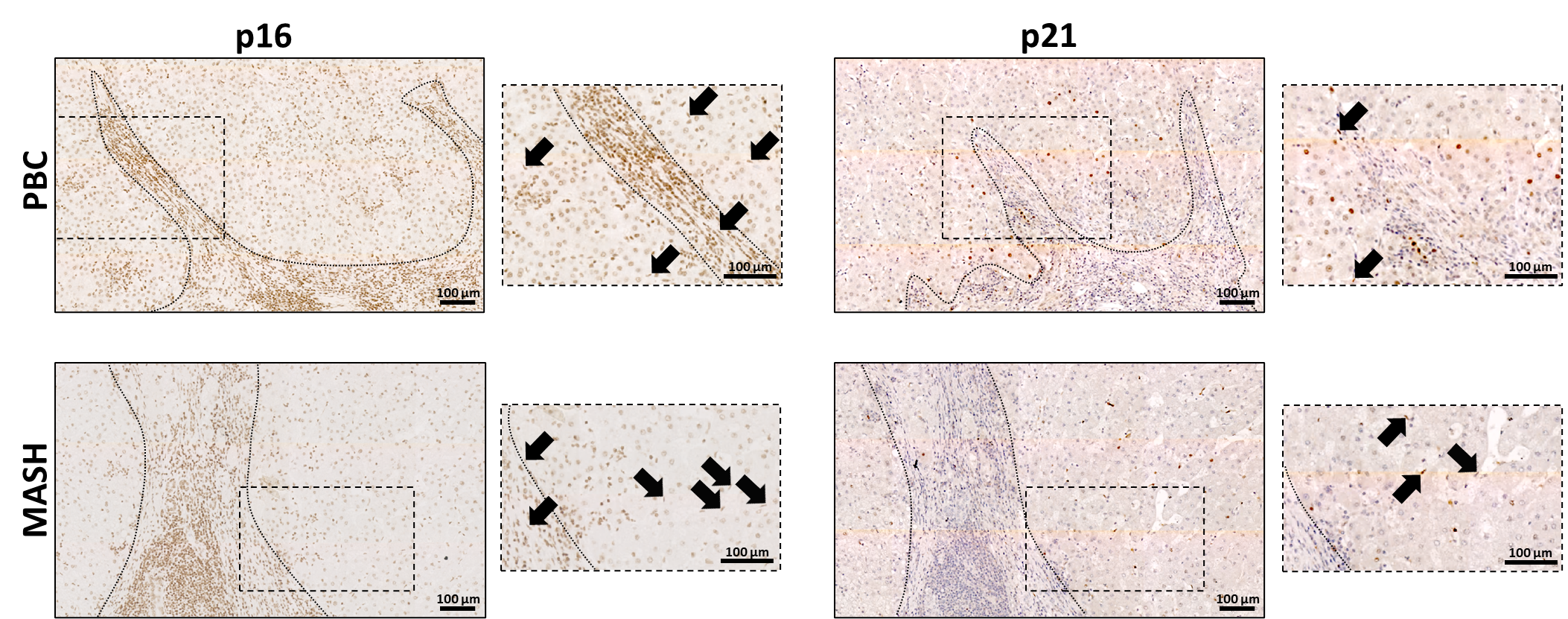


**Supplementary Figure 1 – p16 and p21 staining in chronic liver disease tissues.** Representative images of immunohistochemical staining (brown) for p16 (left) and p21 (right) in chronically diseased liver tissues. PBC = primary biliary cholangitis, MASH = metabolic-associated steatohepatitis. Fibrotic septa are delineated with black dotted line. Black arrows indicate positively stained endothelial nuclei.


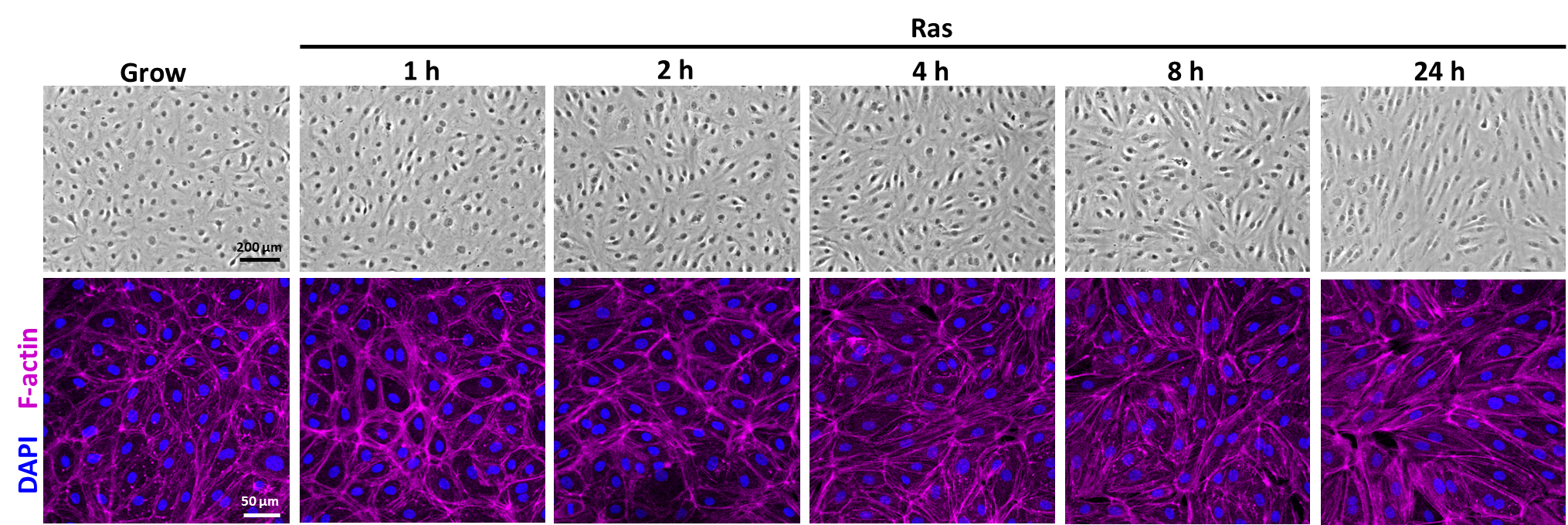


**Supplementary Figure 2 – Time-dependent morphological and cytoskeletal changes in primary human liver endothelial cells in response to acute SASP treatment**. Primary human liver endothelial cells were treated with Grow supernatant for 24 h or Ras supernatant for 1, 2, 4, 8 or 24 h and were fixed in 4% paraformaldehyde (PFA). Following fixation cells were imaged via phase contrast microscopy (*top*) or confocal microscopy (*bottom*). Nuclei were labelled with DAPI (blue) and filamentous actin labelled with phalloidin AF633 (magenta).


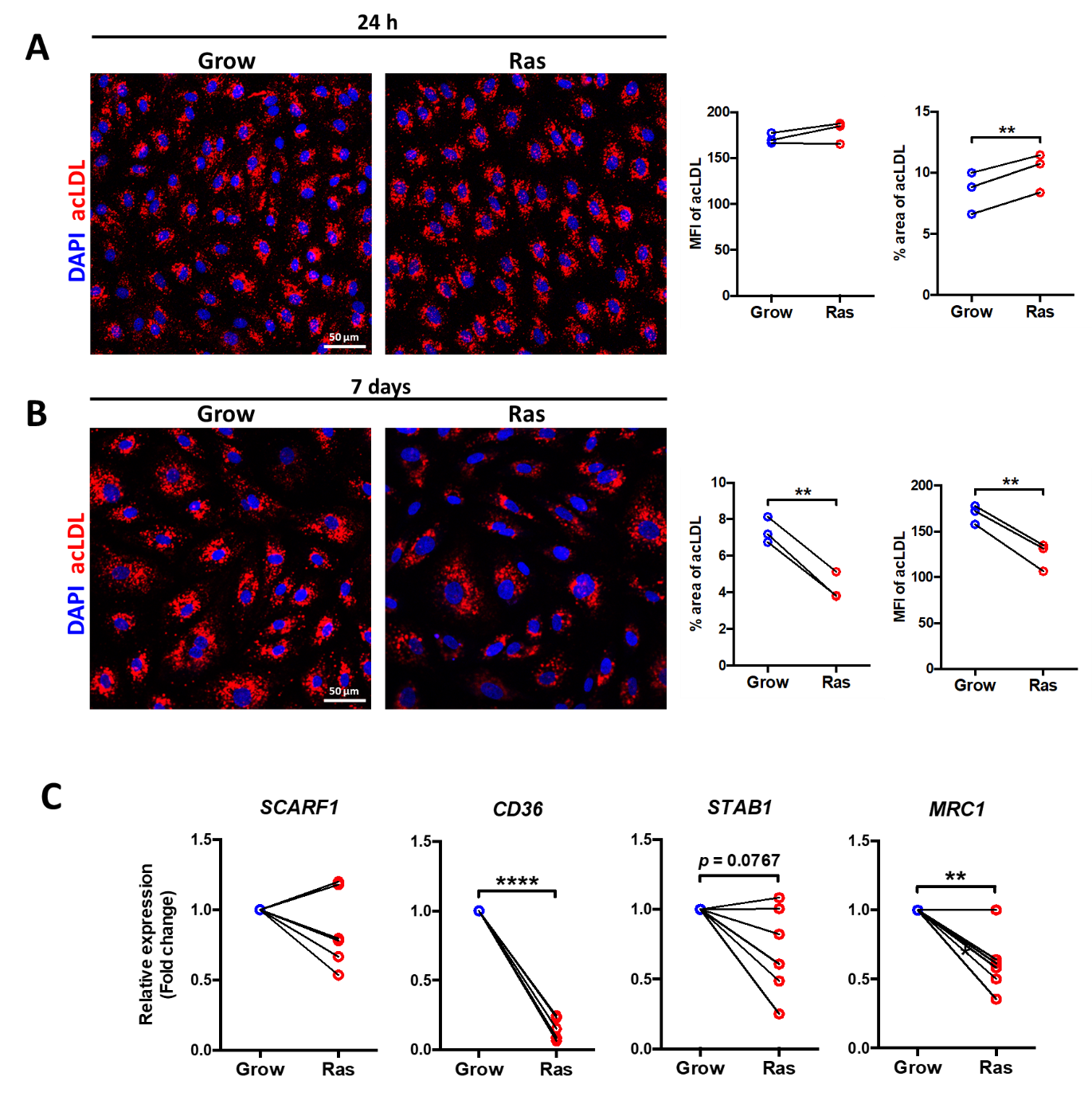


**Supplementary Figure 3 – Altered scavenging capacity of SASP-treated primary human liver endothelial cells.** (A) *Left* Primary human liver endothelial cells pretreated with Ras or Grow supernatants for 24h were treated with 10 µg/ml fluorescently labelled acetylated low-density lipoprotein (Dil-acLDL) for 2h and fixed in 4% paraformaldehyde (PFA). *Right* Mean fluorescence intensity (MFI) and % area positivity were measured. ** indicates statistical significance, where p≤0.01. (B) *Left* Primary human liver endothelial cells pretreated with Ras or Grow supernatants for 7 days were treated with 10 µg/ml fluorescently labelled acetylated low-density lipoprotein (Dil-acLDL) for 2h and fixed in 4% paraformaldehyde (PFA). *Right* Mean fluorescence intensity (MFI) and % area positivity were measured. ** indicates statistical significance, where p≤0.01. (C) qPCR of scavenger receptor genes in liver endothelial cells with SASP (Ras) or growing cell control supernatant (Grow) for 7 days. n = 6 independent donors. ** and **** indicate statistical significance, where p≤0.01 and p≤0.001, respectively.

**
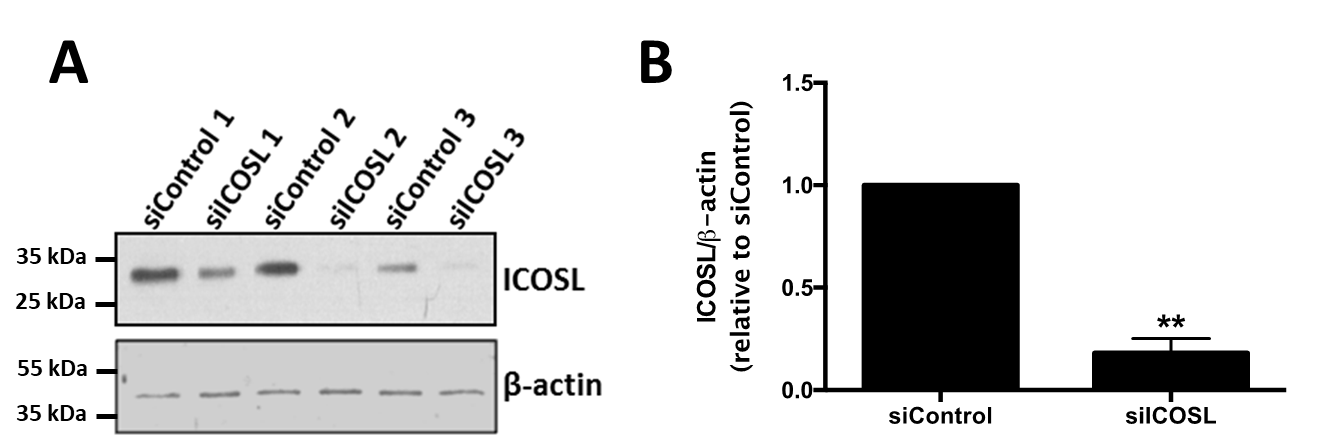
**

**Supplementary Figure 4 – siRNA knockdown of ICOSL in primary human liver endothelial cells.** (A) Representative western blot of ICOSL and β-actin housekeeping protein in primary liver endothelial cells treated with siRNA knockdown of ICOSL (siICSOL) or Silencer™ Select Negative Control (siControl). (B) Quantification of ICOSL expression, normalised to the level of β-actin expression and expressed relative to the Silencer™ Select Negative Control (siControl) treated cells. *n* = 3 independent experiments with different liver endothelial cells. ** indicates statistical significance, where p≤0.01.
