# Supplementary material for: Senescent Human Liver Endothelial Cells Mediate CD4 + T Cell Recruitment via ICOSL
Source: Immunology. 2026 Jun 21;179(1):149–60. doi: 10.1111/imm.70159 (PMC13431846; doi:10.1111/imm.70159)
Supplement: Supplementary file 2 — Data S1: Supporting Information. [file IMM-179-149-s002.docx]

**Immunohistochemistry**

Prior to staining, sections were dewaxed and rehydrated through sequential baths of xylene (x3) and industrial denatured alcohol (IDA) (x3) and distilled water. Antigen retrieval was performed by microwaving in pre-warmed Tris-based antigen unmasking solution (Vector Laboratories) for 20 mins on high power. Slides were slowly cooled to room temperature and incubated in PBS/0.1% Tween^®^ 20 (PBST) for 5 mins. Endogenous peroxidase activity was then blocked with Bloxall^®^ solution (Vector) for 10 mins and blocking of non-specific binding was performed by incubation with 2x Casein Solution (Vector Laboratories, Inc.) for 20 mins. Sections were incubated overnight at 4 °C with anti-p21 primary antibody (2 µg/ml; M7202; Dako) or 1 hour at RT with p16 primary antibody (2 µg/ml; ab54210; abcam), both diluted in PBS. Isotype matched controls at appropriate concentrations were performed in all experiments. Following this, sections were washed twice in PBST for 5 mins and then incubated with the anti-mouse (p21) or anti-rabbit (p16) ImmPRESS™ HRP for 30 mins at RT. Excess secondary antibody was washed off with PBST for 5 mins (twice) and sections were then incubated with DAB chromogen (Vector Laboratories Inc.) for 2 mins; the reaction was stopped with the addition of distilled water. Nuclei were then counterstained with Mayer's Hematoxylin (Pioneer Research Chemicals Ltd.) for 5 mins and slides were washed in warm water for 2 mins. Sections were subsequently dehydrated in sequential baths of IDA (x3) and xylene (x3) and mounted using DPX (Phthalate-free) mounting medium (CellPath). Images were taken using an Axio ScanZ1 microscope (ZEISS).

**Immunofluorescence**

Prior to staining, sections were dewaxed and rehydrated through sequential baths of xylene (x3) and industrial denatured alcohol (IDA) (x3) and distilled water. Antigen retrieval was performed by microwaving in pre-warmed Tris-based antigen unmasking solution (Vector Laboratories) for 20 mins on high power. Slides were slowly cooled to room temperature and incubated in PBS/0.1% Tween^®^ 20 (PBST) for 5 mins. Slides were then blocked for non-specific binding by incubation in PBS with 10% goat serum and 2x Casein solution, for 30 mins at RT. This was followed by 1 h incubation with primary antibodies for p16 (1.25 µg/ml; ab54210; abcam) and CD34 (5 µg/ml; A85659; antibodies.com). Slides were washed twice in PBST followed by 30 mins incubation with Alexa Fluor^®^ conjugated secondary antibodies (1:500 dilution; Thermo Fisher Scientific). Slides were washed and then incubated with Vector TrueView Autofluorescence Quenching Kit (Vector Laboratories, UK, Cat. SP-8400-15) for 5 mins.  Slides were then washed with PBST twice for 5 mins and with 300nM DAPI to counterstain nuclei. Slides were then washed twice with PBST and mounted with VECTASHIELD Vibrance^®^ Antifade Mounting Medium (Vector Laboratories, UK, H-1800-10). Slides were then imaged using a Zeiss LSM 880 confocal microscope.

**Liver endothelial cell isolation and culture**

Briefly, tissues were subjected to mechanical dissociation with scalpels followed by enzymatic digestion via collagenase (10 mg/ml collagenase IA; Sigma-Aldrich). Non-parenchymal cells were separated out via density gradient centrifugation on a 33%/77% Percoll (GE Healthcare) gradient at 800 × *g* for 25 mins. The non-parenchymal cell layer was then removed, and CD45^+^ immune cells and EpCAM^+^ biliary epithelial cells were removed via immunomagnetic selection utilising Dynabeads^TM^ CD45 (Invitrogen) and anti-EpCAM antibody (HEA125) (Progen)/Dynabeads goat anti-mouse IgG (Invitrogen), respectively. Liver endothelial cells were next isolated by positive immunomagnetic selection using CD31 antibody-conjugated Dynabeads^TM^ (Invitrogen). Liver endothelial cells were then seeded in rat tail collagen (RTC) (1 in 100; Sigma-Aldrich)-coated culture vessels in medium composed of human endothelial serum-free media (SFM; Invitrogen) supplemented with 10% human serum (HD Supplies), 10 ng/ml vascular endothelial growth factor (VEGF; PeproTech), and 10 ng/ml hepatocyte growth factor (HGF; PeproTech). Cells were cultured and maintained at 37 °C in a humidified incubator with 5% CO_2_.

**Immunocytochemistry**

For immunofluorescent staining of liver endothelial cells, cells were cultured overnight in rat tail collagen (RTC)-coated μ-Slides VI 0.4 (Ibidi^®^) and treated for 24 h with Ras or Grow supernatants and then fixed in 4% paraformaldehyde (PFA). Alternatively, cells were treated with Ras or Grow supernatants for 6 days and seeded in rat tail collagen (RTC)-coated μ-Slides VI 0.4 (Ibidi^®^) before being fixed in 4% PFA. Following fixation, all cells were washed in PBS, permeabilised with PBS + 0.3% Triton X100 (Sigma Aldrich) for 5 min and then blocked in PBS + 10% goat serum for 20 min. The cells were then incubated at room temperature with anti-CD31 (1.25 μg/ml; A249604; antibodies.com) primary antibody or mouse IgG1 control antibody diluted in PBS for 1 h. The cells were then washed with PBS three times and incubated with appropriate Alexa Fluor^TM^ 488 mIgG1 secondary antibody (1:500; Thermo Fisher Scientific). Filamentous actin was visualised by labelling with Alexa Fluor™ 633 Phalloidin (1 in 40; Invitrogen) for 10 mins, followed by nuclear labelling with 300 nM DAPI for 5 mins. Cells were washed with PBS three times and left in PBS following the final wash before imaging on a Zeiss 880 Zen confocal microscope (ZEISS).

In flow-based adhesion assays (see ‘Flow-Based Adhesion Assays’), liver endothelial cells were treated with Ras or Grow supernatants for 6 days and then cultured overnight in RTC-coated μ-Slides VI 0.4 (Ibidi^®^). Cells were pre-stained with CellTracker^TM^ Green CMFDA Dye (1 in 5000; Thermo Fisher Scientific) in Endothelial SFM + 0.1% BSA (Gibco™ by Thermo Fisher Scientific) for 30 mins at 37 °C. Peripheral blood lymphocytes (PBLs) were isolated from HFE patient blood via Lympholyte®-H (Cedarlane) and pre-stained with CellTrace^TM^ Violet (1 in 5000; Thermo Fisher Scientific) in Endothelial SFM + 0.1% BSA (Gibco™ by Thermo Fisher Scientific) for 30 mins at 37 °C. PBLs were then perfused over liver endothelial cells at a cell density of 1 × 10^6^ cells/ml as described below (see ‘Flow-Based Adhesion Assays’). Liver endothelial cells and adherent PBLs were then fixed in 4% PFA, permeabilised with PBS + 0.3% Triton X100 (Sigma Aldrich) for 5 min and stained with Alexa Fluor™ 633 Phalloidin (1 in 40; Invitrogen) for 10 mins. Nuclear labelling was performed with 300 nM DAPI for 5 mins and cells were washed with PBS three times before being left in PBS prior to imaging on a Zeiss 880 Zen confocal microscope (ZEISS).

**Quantitative real-time (qRT)-PCR**

Briefly, primary liver endothelial cells treated Ras or Grow supernatant for 6 days were cultured overnight in 6-well RTC-treated plates (Corning) and lysed *in situ* with RLT buffer containing 1% β-mercaptoethanol. Following RNA extraction (as per the manufacturer’s instructions), quantity and purity were assessed using a NanoPhotometer (Geneflow), and reverse transcription was performed using the SuperScript III Reverse Transcriptase Kit (ThermoFisher Scientific). qRT-PCR was performed in triplicate to assess mRNA expression using TaqMan Gene Expression Assays (Applied Biosystems) and TaqMan Universal PCR Master Mix (Applied Biosystems). Reactions were performed in a LightCycler 480 (Roche) by completing 40 cycles of the following program: 95°C for 10 s; 60°C for 50 s; 72°C for 1 s. Relative gene expression was calculated following normalisation to the housekeeping gene, *GAPDH*, and data was displayed as fold change to the control.

**Primary lymphocyte isolation**

25 ml of whole blood was layered on 15 ml of Lympholyte^®^-H (Cedarlane) and centrifuged at 800 × g for 25 mins. The peripheral blood mononuclear cell (PBMC) layer was removed and washed in MACS buffer (PBS with 2% FCS (Gibco™ by Thermo Fisher Scientific) and 1 mM EDTA (Gibco™ by Thermo Fisher Scientific)) and centrifuged at 800 × *g* for 5 mins. A platelet depletion step was then performed by a second wash in MACS buffer and centrifugation at 350 × *g* for 10 mins. CD4^+^ and CD8^+^ T lymphocytes were subsequently isolated from PBMCs via negative selection using the Dynabeads™ CD4^+^ T Cell Kit and Dynabeads™ CD8^+^ T Cell Kit, respectively. Manufacturer's instructions were followed for both isolation kits.

**Western blot**

Protein concentration of cell lysates generated from siRNA knockdown of ICOSL in liver endothelial cells was determined by bicinchoninic acid (BCA) assay (Sigma), using bovine serum albumin (BSA) as a protein standard. Aliquots were subsequently diluted to a concentration of 2 mg/mL in CelLytic MT lysis buffer, before storage at −20°C. Protein lysates (20 μg) were separated via SDS-PAGE on 10% acrylamide gels and transferred to 0.2 µm nitrocellulose membranes via the Trans-Blot^®^ Turbo Transfer System (BioRad). Membranes were blocked with 5% non-fat milk solution (Marvel) in PBS + 0.02% Tween 20 (Sigma) (PBS/T) for 1 h at room temperature, before overnight incubation at 4°C with anti-ICOSL primary antibody (1 µg/ml; 16-5889-82; eBioscience^TM^). Following this, membranes were washed three times with PBS/T, followed by addition of a horseradish peroxidase (HRP)-conjugated anti-mouse IgG antibody (1 in 2500; A4416; Sigma-Aldrich) and incubation for 1 h at room temperature. Following three additional wash steps with PBS/T, protein bands were detected using Pierce Enhanced Chemiluminescence (ECL) Substrate (Thermo Fisher Scientific) and visualisation on a ChemiDoc MP Imaging System (BioRAD). Membranes were then stripped with Restore™ Western Stripping Buffer (Thermo Fisher Scientific) for 10 mins, washed in PBS/T twice and re-blocked with 5% non-fat milk in PBS/T for 1 h at room temperature and incubated overnight at 4 °C with the housekeeping anti-β-actin antibody (1 in 2500; A5441; Sigma-Aldrich). Membranes were then washed three times in PBS/T and incubated with HRP-conjugated ant-mouse IgG for 1 h at room temperature. Proteins bands were again detected via ECL.

**Flow-based adhesion assays**

Flow-based adhesion assays over monolayers of primary human liver endothelial cells or HUVECs overexpressing ICOSL were used to study lymphocyte recruitment *in vitro*, under conditions of physiological flow, as described previously (Wilkinson et al., 2024). For 7-day treatment assays, liver endothelial cells were treated with Ras or Grow supernatants for 6 days and approx. 7.5 × 10^5^ cells were seeded in RTC-coated μ-slide VI 0.4 and cultured overnight in RTC-coated μ-Slides VI 0.4 (Ibidi^®^). For ICOSL manipulation studies, approx. 7.5 × 10^5^ of liver endothelial cells or HUVECs overexpressing ICOSL (or vector control cells) were seeded in RTC-coated μ-slide VI 0.4 and cultured overnight. For antibody blockade of ICOSL, liver endothelial cells were treated with Grow or Ras supernatants for 24 h and incubated for 30 mins with anti-ICOSL antibody (10 µg/ml; 16-5889-82; eBioscience^TM^) or mouse IgG1 negative control (10 μg/ml; DAKO) immediately preceding each assay. For siRNA knockdown assays, expression of ICOSL was suppressed before the flow-based adhesion assays were performed and cells were treated with Grow or Ras supernatants for 24 h (see ‘siRNA knockdown of ICOSL in liver endothelial cells’). For assays using HUVECs overexpressing ICOSL (or vector control cells), cells were also treated with Ras supernatant for 24 h. Next, CD4^+^ or CD8^+^ T lymphocytes were isolated (see ‘Primary Lymphocyte Isolation’ above) and resuspended at a cell density of 1 × 10^6^ cells/ml in a flow medium of Endothelial SFM + 0.1% BSA. Lymphocytes were then perfused over the liver endothelial cells at a physiological shear of 0.05 Pa, with each channel of the μ-slide perfused for 5 min. Subsequently, channels were washed though for 3 min with flow medium alone, after which video recordings were taken. All flow assays were imaged via phase-contrast microscopy on an Olympus IX50 Inverted Microscope (Olympus) and 12 fields of view from each channel were analyzed. The number of adherent lymphocytes was firstly counted and then normalized to cells/mm^2^/10^6^ cells perfused using the following equation: adherent cells/flow rate (0.28 ml/min) × bolus (5 min) × field of view area (0.154 mm^2^) × (1/ concentration of lymphocytes 1 × 10^6^ cells/ml).
